# Automatic Coronary Artery Segmentation Using Active Search for Branches and Seemingly Disconnected Vessel Segments from Coronary CT Angiography

**DOI:** 10.1371/journal.pone.0156837

**Published:** 2016-08-18

**Authors:** Dongjin Han, Hackjoon Shim, Byunghwan Jeon, Yeonggul Jang, Youngtaek Hong, Sunghee Jung, Seongmin Ha, Hyuk-Jae Chang

**Affiliations:** Yonsei University, College of Medicine, 134 Sinchon, Seodaemun, Seoul, Korea; Chongqing University, CHINA

## Abstract

We propose a Bayesian tracking and segmentation method of coronary arteries on coronary computed tomographic angiography (CCTA). The geometry of coronary arteries including lumen boundary is estimated in Maximum A Posteriori (MAP) framework. Three consecutive sphere based filtering is combined with a stochastic process that is based on the similarity of the consecutive local neighborhood voxels and the geometric constraint of a vessel. It is also founded on the prior knowledge that an artery can be seen locally disconnected and consist of branches which may be seemingly disconnected due to plaque build up. For such problem, an active search method is proposed to find branches and seemingly disconnected but actually connected vessel segments. Several new measures have been developed for branch detection, disconnection check and planar vesselness measure. Using public domain Rotterdam CT dataset, the accuracy of extracted centerline is demonstrated and automatic reconstruction of coronary artery mesh is shown.

## Introduction

Three-dimensional (3-D) reconstruction of the coronary arteries from coronary computed tomographic angiography (CCTA) would lead to higher accuracy and reproducibility in the diagnosis and to better precision in the quantification of severity of the coronary arteries diseases. It is also essential for the 3-D reconstruction and post-processing tools such as curved multi-planar reformatted (MPR) images through the lumen of each coronary artery. Furthermore, it is also one of the prerequisite steps in subsequent analysis, such as detection of lesions [1] and image fusion [2, 3]. Also, full reconstruction of coronary artery tree with lumen boundary is important.

Since advances in computational fluid dynamics and image-based modeling now permit determination of rest and hyperemic coronary flow and pressure from CCTA, these techniques have been used to non-invasively compute fractional flow reserve (FFR), which is the ratio of maximal coronary blood flow through a stenotic artery to the blood flow in the hypothetical case that the artery was normal, using CTA images [4]. It is pre-assumption that the entire artery tree structure with accurate lumen boundary has to be obtained for the use of such technology.

A significant amount of research has been done on the segmentation of vascular structures and of the coronary arteries in particular [5, 6]. However, many of these vessel segmentation methods require user interaction: manual definition of the start and end points and vessel directions [7], or manual insertion of the intermediate points to bridge gaps [8–10]. Under these interactive segmentation methods, the accuracy of segmentation result is strongly dependent on the interaction from individual users implying a low reproducibility in segmentation results. A trade-off between robustness and automation has to be carried out whenever the segmentation algorithm is to be embedded in a commercial package for clinical applications. Moreover, a certain amount of interaction usually leads to the increment of the processing time.

Several approaches were proposed for automatically tracking the coronary arteries. They appear in various forms. Tek et al. [11] detected ostium locations on the surface of aorta and then started the multi-scale medialness-based vessel tracking from the ostium locations. Medialness filter is their base of vessel finding. Kitslaar et al. [12] uses connected component and morphological filter to detected vessel regions and refines centerlines. Bauer et al. [13] presented an automatic approach that consists of generic methods for detection of tubular objects, extraction of their centerlines, and grouping of these centerlines into tree structures. Zambal et al. [14] calculated candidates for coronary artery seeds and tracks the vessel segments based on vessel surface gradients by approximating the position of the heart and then cylindrical sampling patterns were fitted. Kitamura et al. [15] proposed a method to train a classifier of a tubular 3-D object with a dimension reduction approach using Hessian analysis. Zhou et al. [16] segmented the vascular structures within the heart region using a multi-scale coronary response and then tracked the coronary arteries by a 3-D dynamic balloon tracking algorithm.

The purpose of this work is to extract the whole coronary artery tree segmentation system by actively searching for branches and disconnected vessels. It utilizes the statistical branch occurrence model and checking disconnection model by image appearance. While tracking vessels with the stochastic model for curvature penalized vessel geometry, it finds the branches, a stenosis lesion and seemingly disconnected vessels by atherosclerotic plaque. The whole tree structure is robustly constructed with a probabilistic branch detection method.

## Methods

### Bayesian Formulation of Vessel Tracking Problem

**Fig 1:**
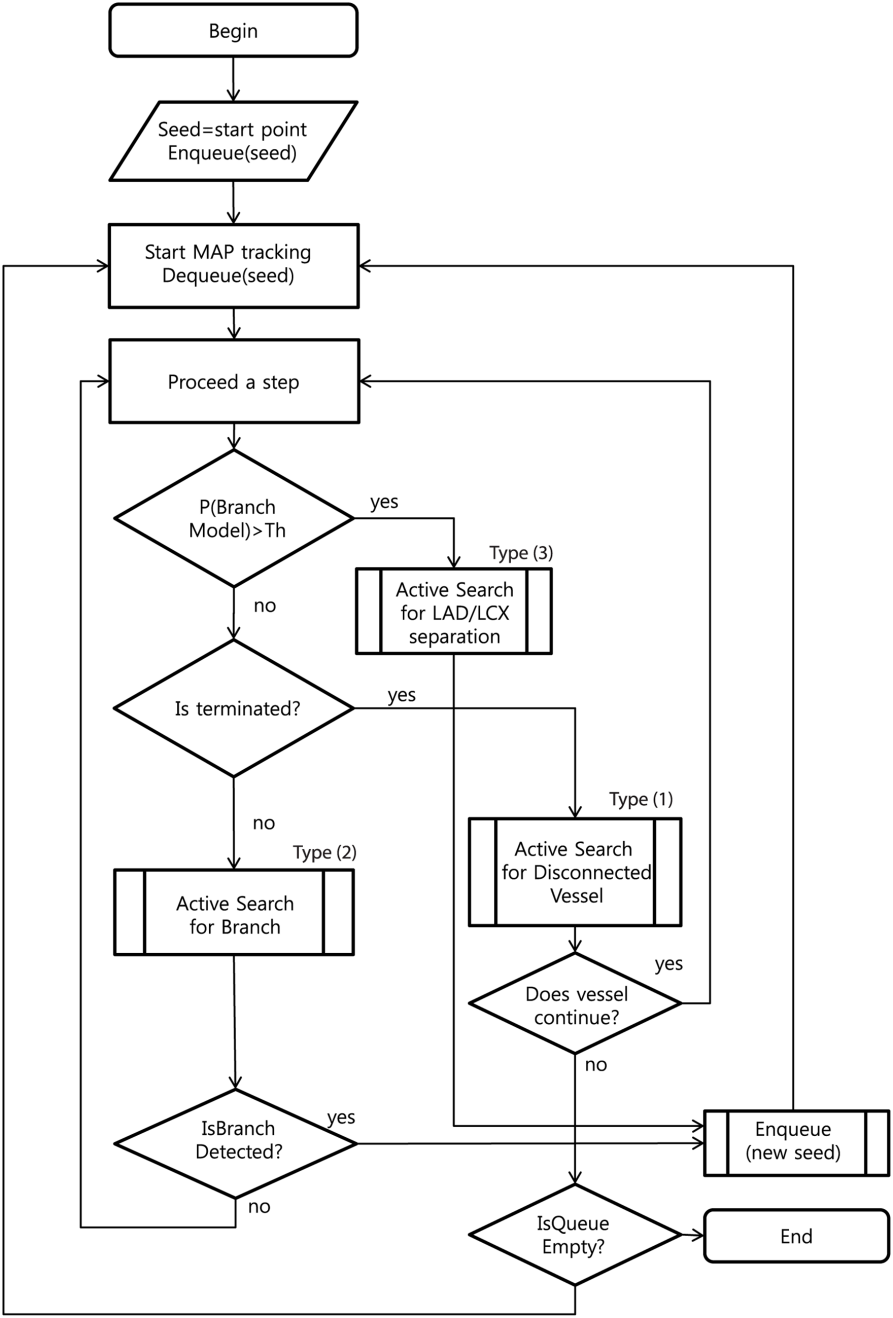
System workflow.

Fig 1 shows the workflow of the proposed method. A three dimensional stochastic process model is built based on vessel shape observation. Autoregressive processes are designed to model vessel geometry.

#### Vessel Tracking as MAP Estimation Problem

Our general framework for vessel tracking and segmentation is to estimate the geometry of the vessel by formulating the problem as MAP (maximum a posteriori) estimation. We look for that vessel for which P(hypothesizedvesselmodel|imagedata) is a maximum. Since this posteriori likelihood of a hypothesized vessel model given the image data can be written as

$$\begin{array}{c} P(\mathrm{hypothesized}\;\mathrm{vessel}\;\mathrm{model}\;|\;\mathrm{image}\;\mathrm{data})\\ =\frac{P(\mathrm{hypothesized}\;\mathrm{vessel}\;\mathrm{model},\;\mathrm{image}\;\mathrm{data})}{P(\mathrm{image}\;\mathrm{data})} \end{array}$$ (1)

and the denominator is not a function of the hypothesized vessel model, the vessel model estimate can be found as that which maximizes the numerator, i.e., the joint likelihood of a hypothesized vessel model and the image data. And the joint probability can be written as

$$\begin{array}{c} P(\mathrm{hypothesized}\;\mathrm{vessel}\;\mathrm{model},\;\mathrm{image}\;\mathrm{data})\\ \propto P(\mathrm{image}\;\mathrm{data}|\;\mathrm{hypothesized}\;\mathrm{vessel}\;\mathrm{model})\\ \times P(\mathrm{hypothesized}\;\mathrm{vessel}\;\mathrm{model}) \end{array}$$ (2)

The vessel geometry at i is denoted *X*_*i*_, $X_{i}={\left(\vec{x_{i}},\vec{r_{i}}\right)}^{\prime }$. $\vec{x_{i}}$ and $\vec{r_{i}}$ represent the coordinate of the center point in 3-D and radius at *i*’th position respectively. Denote by $\underline{\bar{X}}$ the vector having all the *x*_*i*_, *r*_*i*_ in a vessel as its components and by $\underline{\bar{Y}}$ the vector having as its components the CT intensities *y*_*l*,*m*,*n*_ at all the voxels (l, m, n) in the vessel region. Then the proposed system seeks the vessel geometry maximizing the following equation:

$$\begin{array}{c} \max_{\underline{\bar{X}}}P\left(\underline{\bar{Y}}|\underline{\bar{X}}\right)P\left(\underline{\bar{X}}\right) \end{array}$$ (3)

where $P(\underline{\bar{Y}}|\underline{\bar{X}})$ is the likelihood and $P(\underline{\bar{X}})$ is the prior distribution.

#### Geometric Vessel Model

The vessel model is generated based on the following assumptions and statistically trained. The cross-section of a vessel is closed curve with an arbitrary form satisfying the flowing shape restrictions:

1. The vessel radius variation is small and the vessel radius change is likely to be slow.
2. The vessel direction changes are likely to be slow.
3. The local average of gray level in a vessel is likely to vary slowly.
4. The gray level variation between a vessel and background is likely to be large.

Meir [17] uses stochastic model for winding road from satellite image. Their stochastic model reflects faithfully the behavior of long and snaky shape object which narrows and widens along its centerline with the geometric constraint. This model can be easily extended to three dimensional problems. Vessels in human body have similar characteristics as the roads in satellite images. They both change the propagating directions and widths slowly along its centerline with occasional branches. For our problem, a three dimensional stochastic process model is built exhibiting the preceding vessel geometry.

Specifically, autoregressive processes are designed to model vessel center line, vessel width, gray level within the vessel, edge strength at the vessel boundary, and gray levels outside the vessel and adjacent to the boundaries. These regions are referred as background. Note that the vessel geometry processes are hidden, i.e., they are not observed directly in the data. The stochastic processes are functions of a discrete parameter *i* which can be thought of as time or distance, in voxels, along three dimensional axis. Vessel center-curve at *i* is $\vec{x_{i}}$, which takes values in 3-D. This variable is not quantized, it takes arbitrary real values. The $\left\{\vec{x_{i}}\right\}$ process is given by Eq (4), where *ε*_*x*_*i*__, is a zero mean, white, Gaussian driving noise. This process will generate a straight line if *ε*_*x*_*i*__ is zero.

$$\begin{array}{c} \vec{x_{i}}=2\vec{x_{i-1}}-\vec{x_{i-2}}+\varepsilon_{x_{i}} \end{array}$$ (4)

$$\begin{array}{c} \vec{r_{i}}=\vec{r_{i-1}}+\varepsilon_{r_{i}} \end{array}$$ (5)

**Fig 2:**
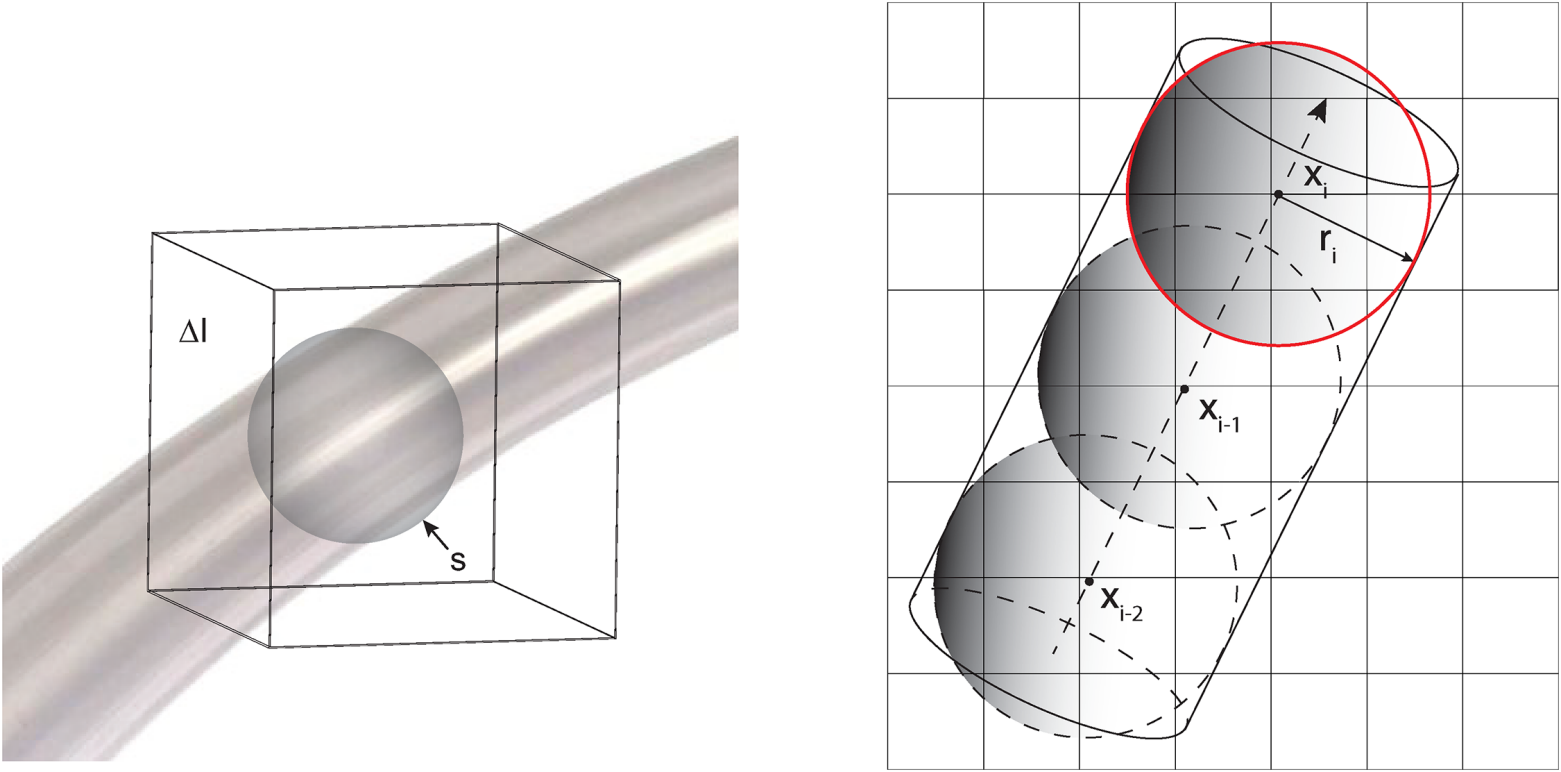
Left: Sphere model for vessel. The foreground is computed inside of sphere *s* and the background is computed using the data in Δ*I* and outside of tube. Right: vessel in a 3-D voxel window without enforced noise.

In Eq (5), vessel radius is modeled such that $\vec{r_{i}}$ is perpendicular circle to center curve $\left\{\vec{x_{i}}\right\}$. The stochastic processes *ε*_*x*_*i*__ and *ε*_*r*_*i*__ are independent Gaussian white noise sequences with zero means and variances *σ*_*d*_*i*__, and *σ*_*r*_*i*__, respectively. The vessel obtained by the un-forced solution(*ε*_*x*_*i*__ = 0, *ε*_*r*_*i*__ = 0) will be straight cylinder with radius *r*_*i*_ as shown in Fig 2. Vessel boundary location is uniquely determined by the $\vec{x_{i}}$ and $\vec{r_{i}}$.

A second order Markov Process is employed to model the mean intensity of the CT image in sequential spherical data of the vessel to be consistent with our vessel model. For foreground and background model,

$$\begin{array}{c} \vec{f_{i}}=\frac{1}{2}\vec{f_{i-1}}+\frac{1}{2}\vec{f_{i-2}}+\varepsilon_{f_{i}} \end{array}$$ (6)

$$\begin{array}{c} \vec{b_{i}}=\frac{1}{2}\vec{b_{i-1}}+\frac{1}{2}\vec{b_{i-2}}+\varepsilon_{b_{i}} \end{array}$$ (7)

$\vec{f_{i}}$ and $\vec{b_{i}}$ are the mean intensities inside and outside of sphere *i*, and *ε*_*f*_*i*__, *ε*_*b*_*i*__ are their Gaussian white noise sequences with zero mean and variance *σ*_*f*_*i*__ and *σ*_*b*_*i*__, respectively.

Here is the list of state variables and equations used in vessel propagation model. General state equation of markov random process can be written for foreground and background as follows.

$$\begin{array}{c} X_{k+1}=[\begin{array}{c} F_{k+1}\\ B_{k+1} \end{array}]=X_{k}+{\vec{N}}_{k}=[\begin{array}{c} F_{k}\\ B_{k} \end{array}]+{\vec{N}}_{k} \end{array}$$ (8)

$$\begin{array}{c} V_{i}=[\begin{array}{c} {\vec{x}}_{i}\\ f_{i}\\ b_{i}\\ r_{i} \end{array}]=V_{i-1}+{\vec{N}}_{i}=[\begin{array}{c} 2{\vec{x}}_{i-1}-{\vec{x}}_{i-2}\\ \frac{f_{i-1}+f_{i-2}}{2}\\ \frac{b_{i-1}+b_{i-2}}{2}\\ r_{i-1} \end{array}]+[\begin{array}{c} \sigma_{x_{i}}\\ \sigma_{f_{i}}\\ \sigma_{b_{i}}\\ \sigma_{r} \end{array}] \end{array}$$ (9)

In Eq (9), the first row gives geometric constraint which comes from Eq (4), and the second and third rows of the equation come from Eqs (6) and (7). They all together sum up to the following equation where maximum-likelihood estimate is found. Furthermore, MAP estimate ${\hat{x}}_{i,r}$ can be also obtained if proper prior is found for vessel geometry.

$$\begin{array}{c} {\hat{x}}_{i,r}=\arg_{i,r}\min\sum_{j=1}^{N}{\left|\right|x_{i}-2x_{i-1}+x_{i-2}\left|\right|}^{2}/\sigma_{x_{i}}\\ +{\left|\right|f_{i}-\left(f_{i-1}+f_{i-2}\right)/2\left|\right|}^{2}/\sigma_{f_{i}}+{\left|\right|b_{i}-\left(b_{i-1}+b_{i-2}\right)/2\left|\right|}^{2}/\sigma_{b_{i}} \end{array}$$ (10)

#### Curvature and Torsion

In differential geometry, the fundamental theorem of curves states that any regular curve in three dimensions with non-zero curvature has its shape completely determined by its curvature and torsion. Curvature measures how much a curve bends on a plane and torsion measures how much a curve deviates from its osculating plane. The shape of a vessel does not change abruptly. Therefore, for curve reconstruction problems, it makes sense to regularize the solution with curvature and torsion prior [18]. If the reconstructed curve should follow closely the shape of an underlying surface which is locally planar. In such situations, it may be advantageous to penalize torsion. As shown in Eq (10), the vessel trajectory is estimated using the paths with two previous points *f*_*i*−1_ and *f*_*i*−2_. In each step of tracking procedure, three points are used for computing likelihood. In other words, the curvature of vessel trajectory is obtained with three points and the curvature constraint is used in our geometric model depending on *ε*_*x*_*i*__ in Eq (4). If *ε*_*x*_*i*__ is small, it will prefer smoother trajectory and if big, it will allow more complex trajectory. Curvature and torsion regularization method for curves is extensively performed in [19, 20]. However, torsion is not currently used in our vessel tracking process. The vessel trajectory model may be improved by employing torsion constraint.

#### Usage of Region Growing in our Method

Region growing is an approach to image segmentation in which neighboring pixels are examined and added to a region class. It is one of the practicable ways to achieve image segmentation and many conventional algorithms in medical image analysis employ region growing method. Region-growing algorithms turn out to be very practicable way to achieve a time-saving vessel segmentation even with the disadvantages due to their inherent sequential nature. However, in vessel segmentation problem, by just region growing implementation, highly accurate vessel segmentation cannot be achieved since many other regions next to vessel have similar HU level, *e.g.,* left ventricle or other vascular organs. It has to be incorporated with other processes to obtain successful vessel segmentation. To overcome such disadvantages, we merely use region growing as a rough guide to reduce the number of computation. Later our geometric vessel model will separate vessels from background correctly.

**Fig 3:**
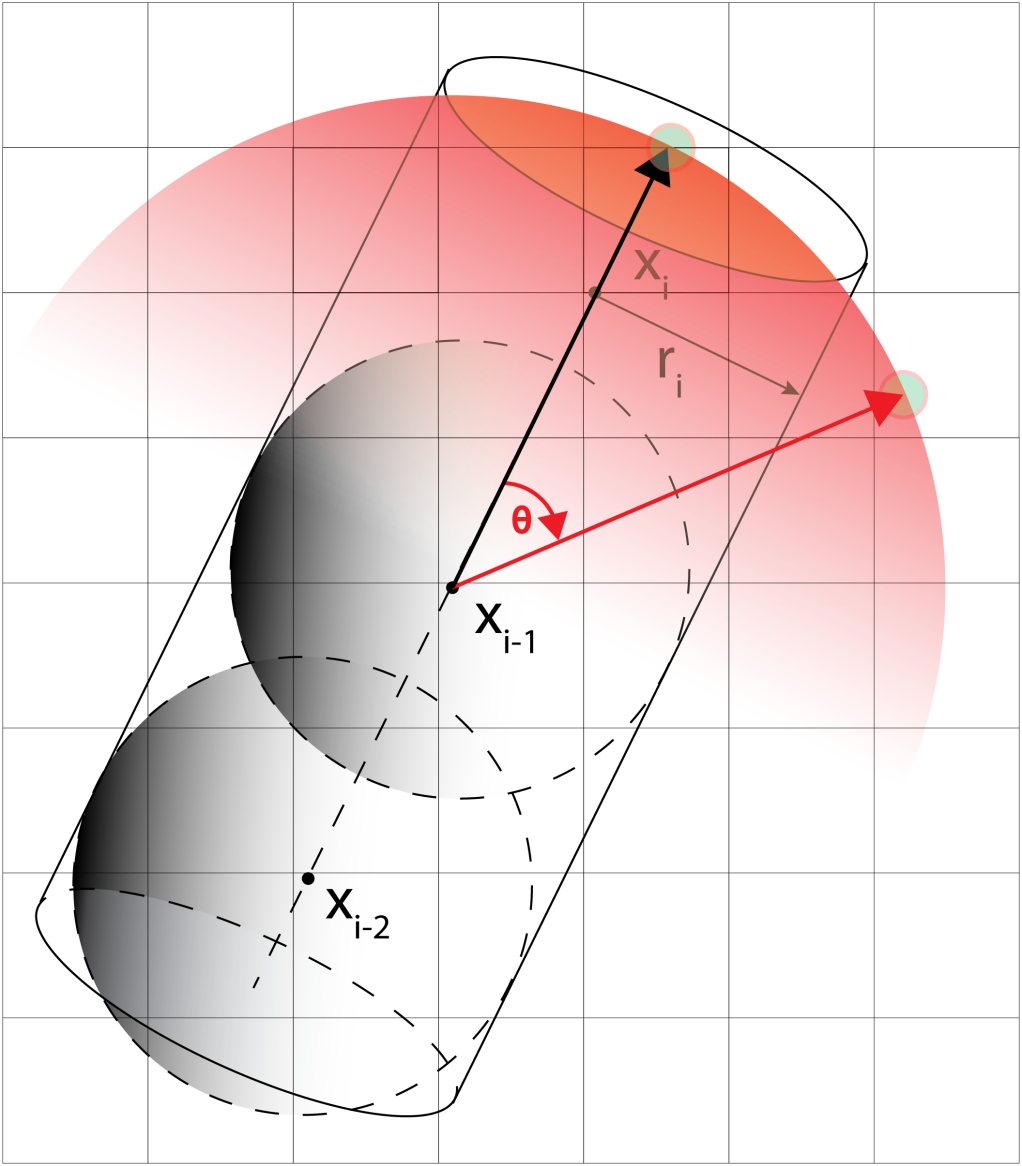
A vessel in a 3-D voxel window with directional constraint.

Adaptive Region growing first selects the candidate region where vessel may lie in. This practical procedure defines the region where the probability of vessel is almost zero and reduces the amount of computation. Fig 3 shows the region growing and vessel geometrical model together. The region growing selects the candidate area (in red) in advance.

### Branch Detection

For the reconstruction of complete coronary artery tree, the tracking or segmentation system requires to find the branches of vessels and track the sub-branch vessels. When a new branch is encountered while tracking a vessel, the tracker must initiate a new vessel seed for tracking and branch detection mechanism is required. However, patients with stenosis occasionally show disconnected vessels in CT due to narrowing or blockage of the coronary arteries. It often occurs near branch points [21]. Hence we devise a method named “active search” which can search and find such disconnected arteries and branches.

For vessel branch detection, conventional methods can be grouped into two categories; region growing based methods and statistical analysis based methods of the vessel propagation distribution. And there are also variants of two categories. For region growing methods, Tek et. al. [11] proposed a method that after extracting centerlines of the three major coronary arteries, the algorithm starts to trace side branches. First, the bifurcation of a side branch is detected on a major centerline using region growing based lumen segmentation. Starting from a centerline point, bright voxels connected to the current point are added iteratively. The growing front is traced. If a side branch presents, the region growing procedure goes into this side branch. A side branch is detected when it finds a front with a distance to the existing major centerline larger than a threshold. At each detected bifurcation point, a data-driven centerline tracing process is initialized. Jiang [22] adopts a sphere propagation method to determine the coronary artery branches. By using different size of sphere and uniform ray casting they find the branch points. They produce incremental size spheres R+1, R+2 and R+3 and propagate uniformly distributed rays. If rays reach outermost shell satisfying pre -specified condition and concentrate at separate two clusters, they can find side branches. The progressive region growing method has been developed and skeletonization method is used for tree structure finding [23]. Such analysis methods are supported by extracting the skeletons or medial axes of the segmented voxel sets.

For statistical method for branch detection, Dip test has been used [24, 25]. A Dip statistic test is to test the multi-modality of the re-sampled particles against the null hypothesis of a unimodal distribution. The Dip test, a measure of departure from uni-modality in 1D, measures the maximum distance between the empirical distribution and the best fitting unimodal distribution. Then, if a junction is detected by using dip test, k-means clustering (k = 2) is performed to find the direction of the daughter branches.

#### Our Branch Detection Procedure

We devise a robust branch detection system incorporating conventional statistical method and newly developed active search method. As shown in Fig 1, while tracking artery, the system continuously detects bifurcation or multifurcation by checking if there exists separate clusters for high probability candidate points. If a cluster or multiple clusters are found, those points are denoted as branch points. For active search scheme, the branches are actively searched in three categories. They are discussed in next subsection.

### Active Vessel (Branch) Search

In CCTA images, vessel sometimes can be seen to be disconnected due to plaque build-up or noise and sequential tracking methods will stop since there is a gap between vessel segments. In such cases, the active search method will overcome the gap.

**Fig 4:**
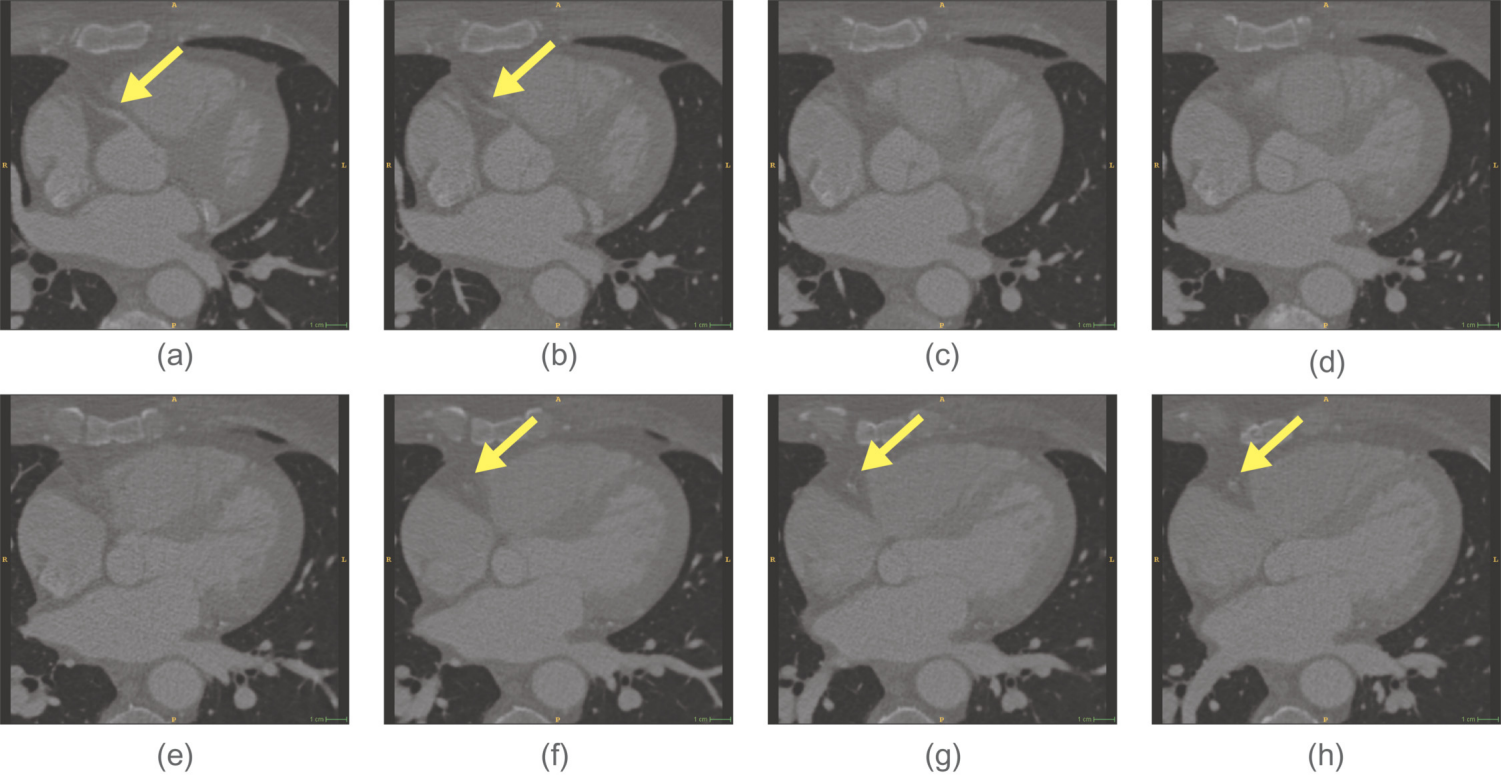
Noisy image makes a vessel appears to be disconnected.

**Fig 5:**
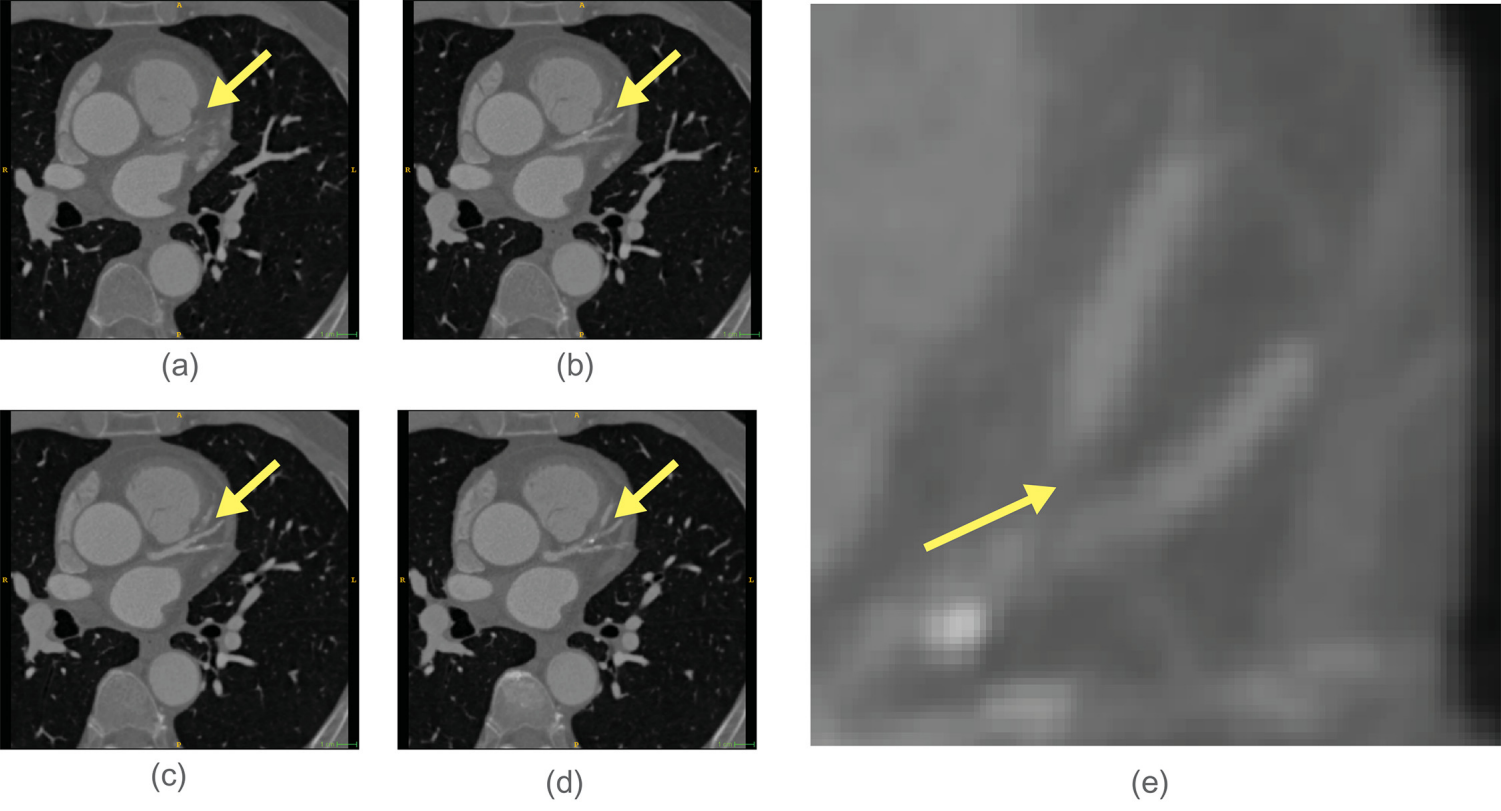
Discontinuity in LAD (left anterior descending) artery shown. (e) is a magnified version of (d).

**Fig 6:**
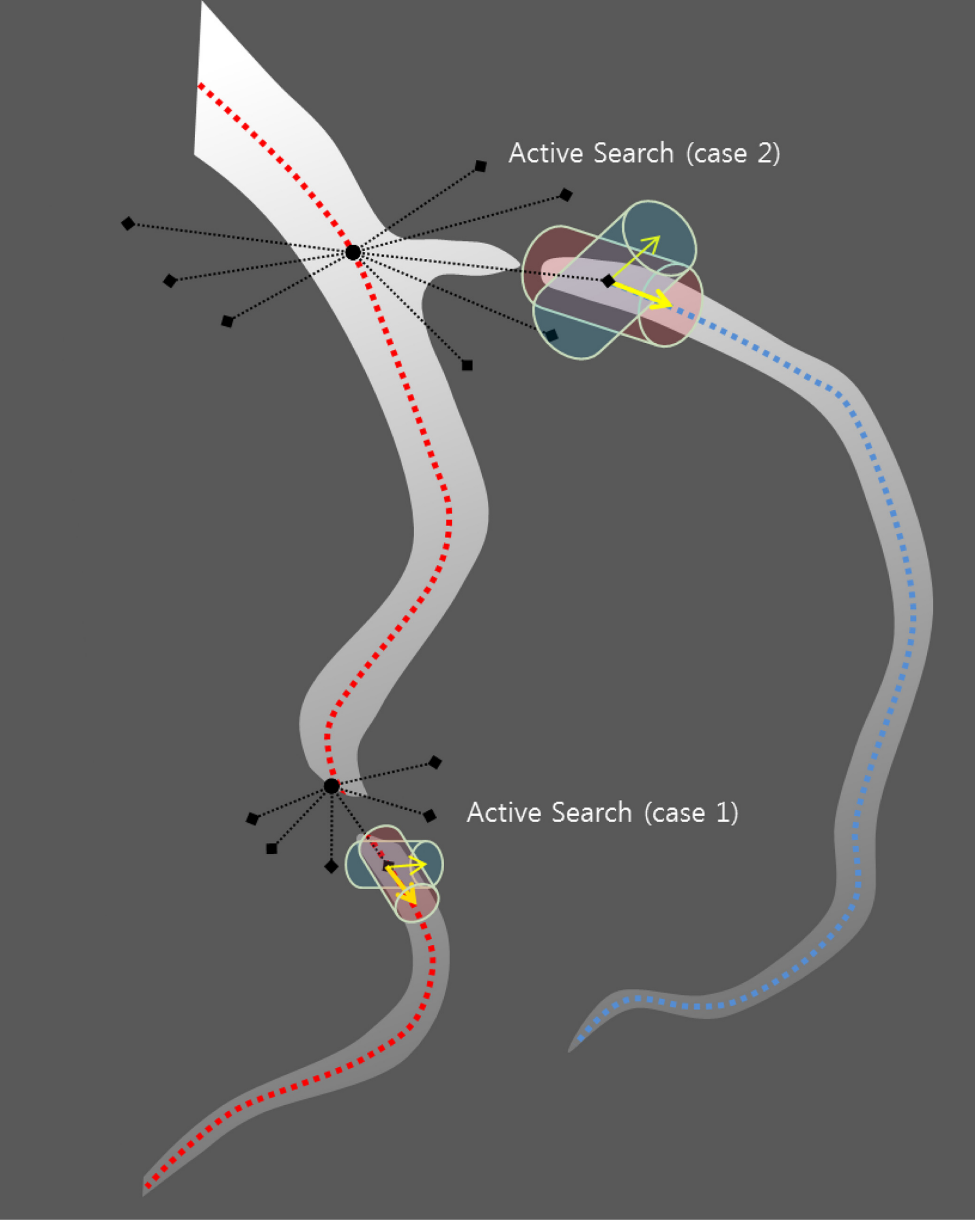
Active search for vessel candidate. Type 1 and 2 active search methods are illustrated. Type 1 is used for disconnected vessel and type 2 is used for disconnected branches.

**Fig 7:**
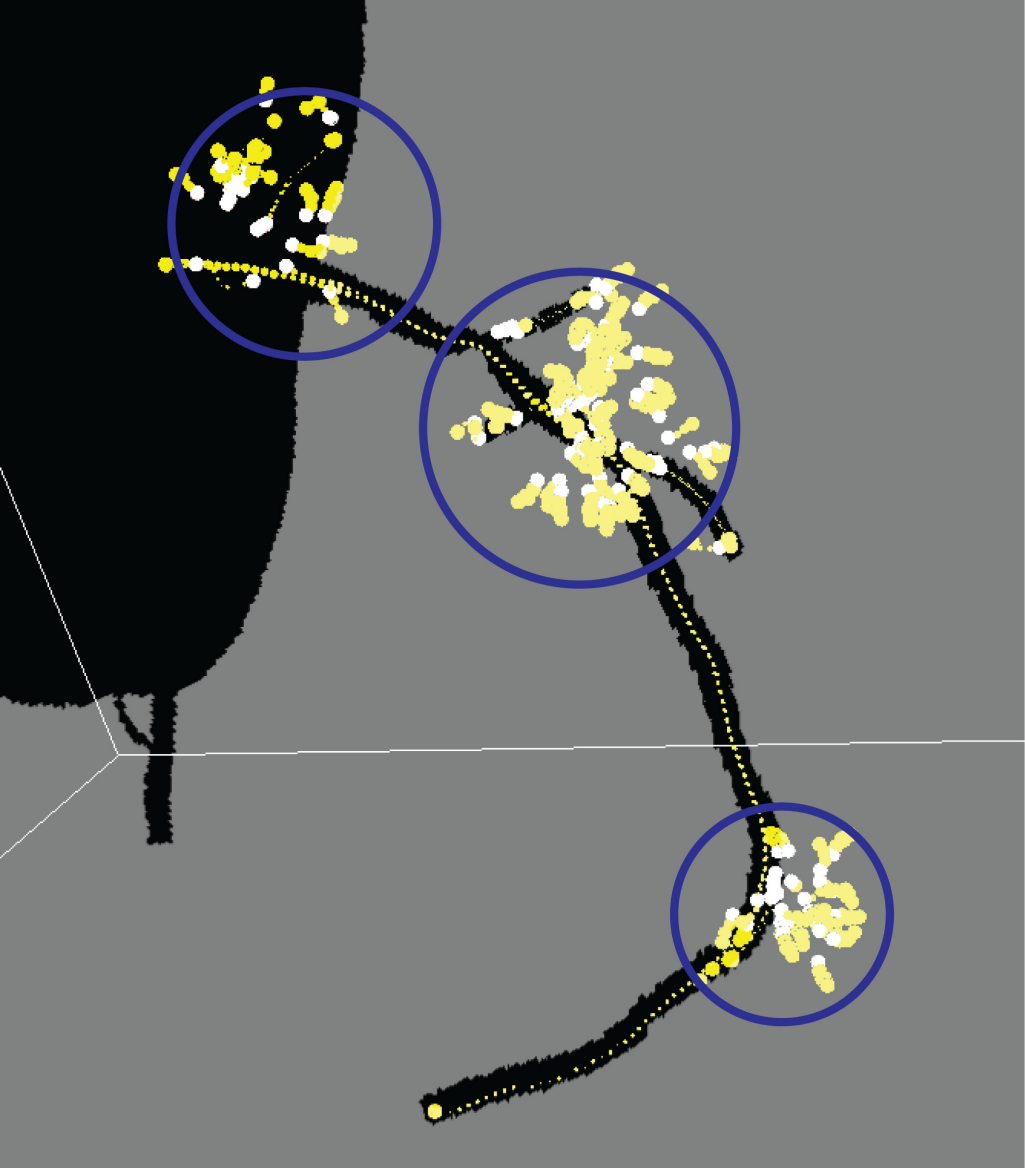
Example of tracked centerlines and actively searched region. Actively searched regions are marked in blue circles. Note there are many false positive seed points (white and yellow spheres). They are automatically deleted later.

The active search are used: to check if a vessel is locally disconnected (type 1), to find disconnected branch (type 2) and according to branch occurrence model (type 3). Their usage is shown in the workflow chart in Fig 1. When a vessel is abruptly disconnected or there is probable branch area, active search is employed. Conventional methods for branch detection described in the above sub-section utilize the fact that sub-branch pipelines are locally detectable and well separated. They test if there is a separation of highly contrasted regions using either region growing or statistical test for probability distributions of tracking mode. However, when local discontinuity exists such methods will fail. This active search method is also applied for branch detection. The examples of both cases follow. In Fig 4 which is one of Rotterdam test data (testdata 26), a vessel is disappearing and re-appearing along several axial slices of images. In the figure, RCA is clearly shown in (a-b) then in (c-e) it is lost and re-appeared again in (f-h). In this case, coronary artery tracking method will stop due to low intensity of invisible vessel. Hence an active search will start when abrupt termination is detected. In other hands, Fig 5 shows a discontinuity at LAD branch point. Near a bifurcation or branching point, discontinuity occasionally occurs due to complex fluid turbulence. However, the active search is an exhaustive method and brute force search will require of a large number of computation. To reduce the number of computation, the branch occurrence model has been devised. The active search comes from three different manners as described above. The following is the description for the local image intensity based active search (type 2). The model is shown in Fig 6 and the real result of the active search (type 2) is shown in Fig 7.

1. Eq (10) gives the likelihood of each direction (point ${\vec{x}}_{i}$). Many points will have high probabilities and cluster into vessel propagation directions. From the cluster of such points, the branch can be detected. If there exists two significant clustering, bifurcation is found. (So far, this is closely related to conventional branch detection DIP test method).
2. If no branch point is found, active search is employed at highly probable region. (i.e., if the neighboring volume **S** has higher intensity above some threshold.)
3. Active search will investigate equally spaced points $\vec{s}$ in the neighboring volume **S** ($\vec{s}\in S$), where *dist*($\vec{s}$, $\vec{x_{i}}$) ≤*T*_*th*_ and $dot\left(\vec{s}-{\vec{x}}_{i},{\vec{x}}_{i}-{\vec{x}}_{i-1}\right)\leq T_{\theta }$ where *dist* is distance measure of two points and *dot* is dot product of two vectors.
4. If new points are found, they will be queued as new seed points.

#### Branch Occurrence Model using Possion Distribution

This type of active search (type 3) is used for ensuring that left main (LM) artery’s first bifurcation is not missing. For the branch occurrence model, the first branch location of left main (LM) artery is measured from the aorta. LM coronary artery usually bifurcates into left anterior descendent artery (LAD) and left circumflex artery (LCX) branches. The distances of this first branch of left main coronary arteries from ostium are shown in Table 1. The training set of Rotterdam data is used for the distance measure.

**Table 1:** Dataset used for Poisson parameter estimation. Poisson pdf ($\hat{\lambda }=17.3$) trained on LM (left main) artery length.

dataset nr.	0	1	2	3	4	5	6	7
first branch location (mm)	21.00	21.96	17.94	17.64	10.86	22.38	15.69	11.19

The occurrence based process can be modeled using Poisson process. Poisson is a discrete probability distribution that expresses the probability of a given number of events occurring in a fixed interval of time and/or space if these events occur with a known average rate and independently of the time since the last event. Poisson distribution is written as

$$\begin{array}{c} P(x)=\frac{e^{-\lambda }\lambda^{x}}{x!}. \end{array}$$ (11)

In Eq (11), *x* is the distance from the ostium and the λ is the expected distance value. Using medical expert annotated distances of first branch from ostium in Rotterdam training data [26, 27], the Poisson distribution model has been trained as shown in Table 1. which shows the distance of first branch from the ostium. $\hat{\lambda }$ is the estimate of Poisson parameter and 17.3325. In the experiments, all of the first branch (LM toward LCX and LAD) were correctly found in 32 dataset by utilizing the active search method together with the Poisson branch occurrence model.

As described before, highly stenotic coronary arteries appear sometimes as disconnected as shown in Figs 4 and 5 even though they are connected. In such cases, we use active search scheme which can detect disconnected vessel and connect them. While MAP estimate is found via maximizing using Eqs (2) and (10), active searching scheme runs at the same time as shown in Fig 1. With two previous center point *x*_*i*−2_, *x*_*i*−1_ and vessel geometry constraint, the prior probability distribution of vessel geometry is assigned. Search candidates points *x*_*i*_’s are uniformly generated on the hemisphere with radius *S* (step size), and each point *x*_*i*_ is assigned with the prior probability depending on geometric constraint. Also the likelihood from foreground and background model and others is computed. The posterior probability is computed from the prior and the likelihood. For branch detection, the candidate points with relatively high posterior probability on the hemisphere is now clustered into small group and the distance of clusters are measured. If the distance is over some threshold the branch is detected. At the same time, active search with Poisson process for the branch occurrence model and active search for high probable region from the HU level is also investigated.

### Planar Vesselness Measure

It is necessary to analyze the shape of coronary artery for determining if detected or segmented object is coronary arteries and not others. While the tracking system tracks down the coronary arteries and their branches, the tracker may stray into wrong region like left ventricle. Tracker needs to check if the tracked object is a vessel. Hence, detection and calculation of topological features for such 3-D tube-like objects are required via description of shapes and other characteristics of complexity. This should allow to represent interior structure of 3-D objects.

The following planar vesselness measure is used in our system. We use a fast and efficient planar version of vesselness measure. It can tell when a vessel tracker must stop or if searched point is a vessel-like point in the active search process.

#### Analysis of Cross Sectional Shape

To speed up the tracking process, simple planar Hessian based measure is employed in our system. To detect foreground objects solely, Frangi proposed a method that can find elongated, i.e., tube-like objects [28] and the method shows successful results. However it requires an extensive 3-D computation along the scale space in conventional Frangi’s vesselness measure. A simple planar version is devised here.

If the planar measure scores low in consecutive cross sections. One can tell it is not a vessel. The underlying assumption is that the cross section of coronary artery or its area is elliptic and has higher HU value compared to myocardium or surrounding materials. Let the linear scale-space representation of the cross sectional image *I*_0_(*x*, *y*) at scale *σ* given by:

$$\begin{array}{c} I\left(x,y;\sigma \right)=I_{0}\left(x,y\right)*G\left(x,y;\sigma \right) \end{array}$$ (12)

where *G*(*x*, *y*; *σ*) is the Gaussian kernel with scale factor *σ*. It starts with iso-intensity surface definition which is

$$\begin{array}{c} I(x+\Delta x,y+\Delta y)=I(x,y) \end{array}$$ (13)

where *σ* is omitted for simplicity. Let *δ* = (Δ*x*, Δ*y*) and using Taylor’s expansion in vector notation, it can be simplified as:

$$\begin{array}{c} \frac{1}{2}\delta H\left(x,y\right)\delta^{T}+\left(I_{x},I_{y}\right)\delta^{T}=0 \end{array}$$ (14)

where

$$\begin{array}{c} H\left(x,y,z\right)=(\begin{array}{cc} I_{xx} & I_{xy}\\ I_{yx} & I_{yy} \end{array}) \end{array}$$ (15)

is the Hessian matrix. This quadratic equation represents a general quadric surface including ellipsoids. Eq (14) can be divided into two parts:

$$\begin{array}{c} \frac{1}{2}\delta H\left(x,y\right)\delta^{T}+\left(I_{x},I_{y}\right)\delta^{T}>0\;\;or<0 \end{array}$$ (16)

If *H* is positive or negative definite, it represents convex or concave region, respectively (*i.e.*, dark area or bright area). Since we are looking for coronary arteries in CCTA, negative definite will be a good indicator.

Therefore, in 2-D cross sectional views of coronary arteries, the Hessian will be $H=(\begin{array}{cc} I_{xx} & I_{xy}\\ I_{yx} & I_{yy} \end{array})$ and the bright elliptic feature like cross section of a vessel is equvallently represented as the set of

$$\begin{array}{c} \left\{\left(x,y\right)|H\left(x,y\right)<0\right\}=\left\{\left(x,y\right)|I_{xx}+I_{yy}<0\cap I_{xx}I_{yy}>I_{xy}^{2}\right\} \end{array}$$ (17)

**Fig 8:**
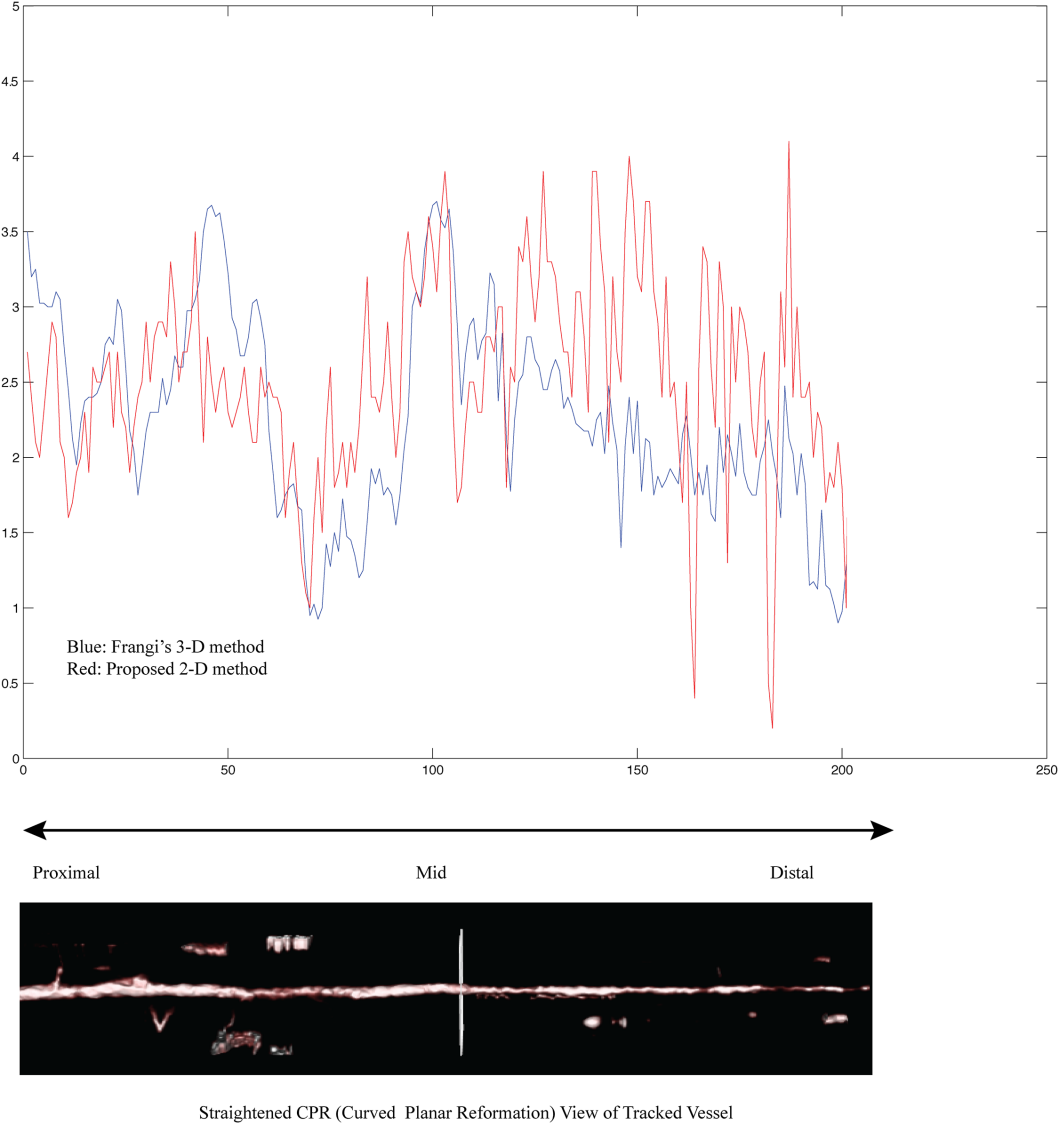
Planar vesselness (red) vs. conventional 3-D Frangi’s Vesselness (blue) along the vessel centerline. Both have high values along the tracked vessel. And they are much correlated at proximal and mid-part of coronary artery. When there is no vessel, both values are near zero.

Hence the planar vesselness measure **v** is computed from Eq (16). It can be written as the function of *I*_*xx*_+*I*_*yy*_ and $I_{xx}I_{yy}-I_{xy}^{2}$. For detail, please refer to [29]. The performance of our planar vesselness measure is compared to conventional 3-D vesselness filter in Fig 8. Both measure have significantly high value along the tracked vessel (especially proximal and mid part of a vessel) and seems to be correlated in the figure. When there is no vessel, both value will be almost zero. The following subsection summarizes the overall branch detection procedure.

#### Initiation of Seed Point for Tracking

**Fig 9:**
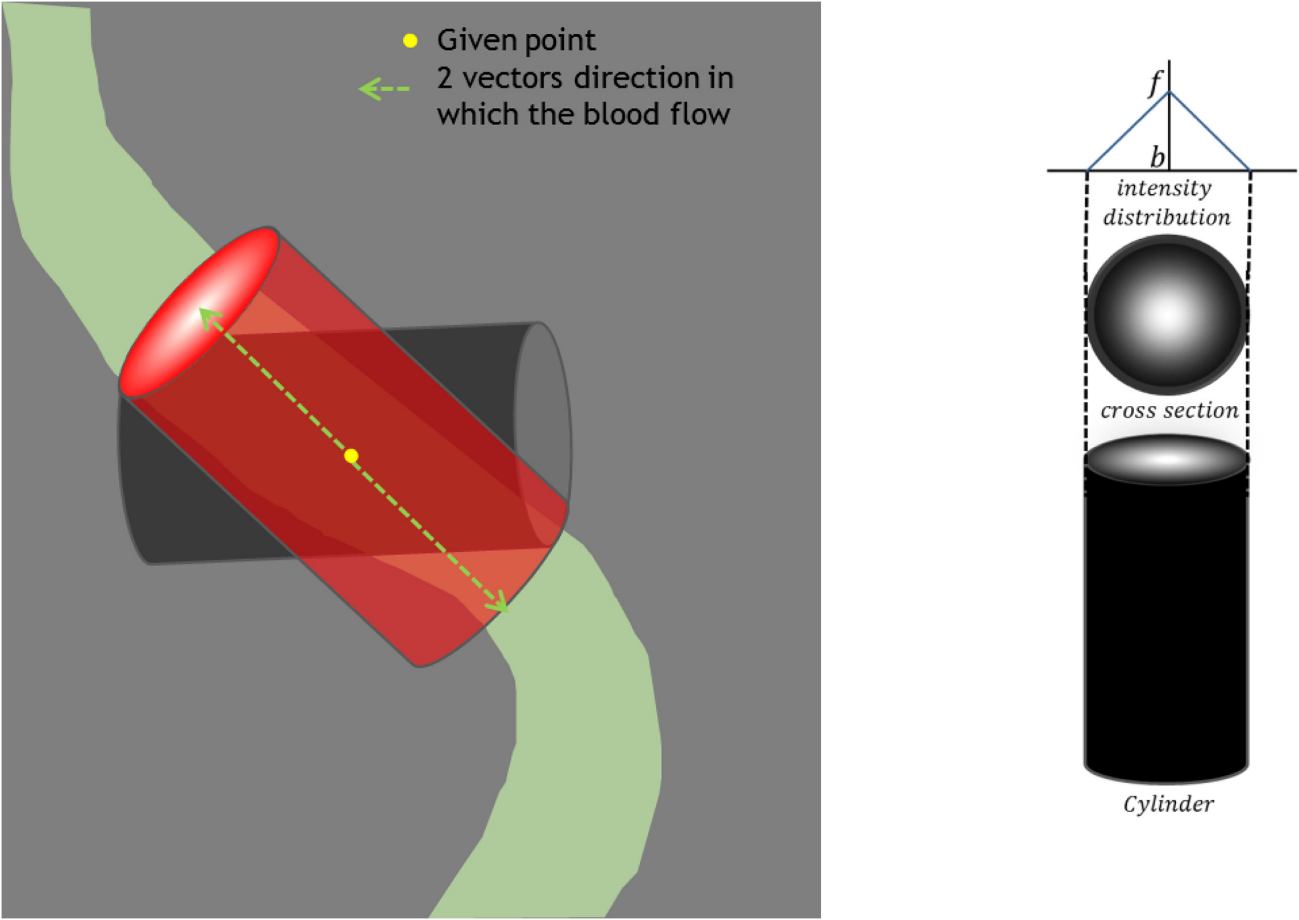
Exhaustive search for initial direction of vessel using cylinder model.

In the experiment, the start points (seed points) are fixed and given in rotterdam coronary artery algorithm evaluation framework. The initial direction is found via ML estimators like the cylinder in Fig 9. At a given seed point *SP* and searching direction in 3-D, the cylindrical mask is generated using Gaussian shaped kernel along the long axis. The most probable direction is found. When active search are used, the initial direction of the vessel is found the same way.

## Experiment

The accuracy of the centerline extracted from the suggested method is tested on 32 CCTA public Rotterdam challenge dataset [26, 27]. These datasets were provided by the organizers of the “Coronary Artery Tracking Challenge”. On the evaluation framework, it measures the accuracy of the centerlines of four significant vessels for each dataset and in total the number of tracked vessel is 128. While the evaluation method only assesses the estimated centerlines, our system finds the centerlines and automatically reconstructs the vessel lumen boundary mesh at the same time. Our centerline extraction experiment using Rotterdam dataset will be categorized as method #2 following the Rotterdam coronary artery algorithm evaluation framework rules. One single point *S* (starting point at ostium) is pre-defined at the challenge site [26, 27] for each left main artery (LM) and right coronary artery (RCA). The detailed experiment procedure can be found at [26]. The result is shown in Tables 2–5. It shows a successful result in overlap measure (OV), overlap until first error (OF) and overlap with clinically relevant vessel (OT) (OV 81.4%, OF 77.3%, OT 87.8%) for 8 training dataset (Table 2) and OV 84.3%, OF 61.2%, OT 88.3% for 24 test dataset (Tables 3–6). Finally, all together with 32 dataset, the result is OV 83.6%, OF 65.3%, OT 88.2%.

**Table 2:** Test result using 8 Rotterdam Training dataset. It shows detailed values for each vessel. (sc: score.).

Dataset nr.	Vessel 0				Vessel 1				Vessel 2				Vessel 3			
0	OV	0.88	sc	44.45	OV	0.97	sc	48.56	OV	1.00	sc	100.00	OV	0.88	sc	44.50
	OF	0.14	sc	7.34	OF	1.00	sc	100.0	OF	1.00	sc	100.00	OF	0.79	sc	40.21
	OT	0.88	sc	44.18	OT	1.00	sc	100.0	OT	1.00	sc	100.00	OT	0.88	sc	44.32
1	OV	0.89	sc	45.01	OV	1.00	sc	62.42	OV	0.86	sc	43.21	OV	0.97	sc	49.02
	OF	0.89	sc	45.04	OF	1.00	sc	100.00	OF	1.00	sc	100.00	OF	1.00	sc	100.00
	OT	0.89	sc	44.74	OT	1.00	sc	100.00	OT	1.00	sc	100.00	OT	1.00	sc	100.00
2	OV	0.90	sc	45.15	OV	0.95	sc	48.02	OV	0.89	sc	44.92	OV	0.21	sc	10.37
	OF	0.89	sc	44.94	OF	0.91	sc	46.22	OF	0.84	sc	42.71	OF	0.00	sc	0.00
	OT	0.90	sc	44.87	OT	0.95	sc	47.73	OT	0.98	sc	48.91	OT	0.40	sc	20.01
3	OV	0.70	sc	35.29	OV	0.58	sc	28.99	OV	0.46	sc	22.98	OV	0.79	sc	39.96
	OF	0.70	sc	35.31	OF	0.41	sc	20.61	OF	0.30	sc	15.12	OF	1.00	sc	100.00
	OT	0.75	sc	37.25	OT	0.59	sc	29.36	OT	0.46	sc	22.84	OT	1.00	sc	100.00
4	OV	0.84	sc	42.02	OV	0.94	sc	47.17	OV	0.78	sc	39.04	OV	0.85	sc	42.71
	OF	0.92	sc	46.49	OF	0.89	sc	45.29	OF	0.65	sc	32.76	OF	1.00	sc	100.00
	OT	0.85	sc	42.74	OT	0.94	sc	47.09	OT	0.79	sc	39.46	OT	1.00	sc	100.00
5	OV	0.69	sc	34.94	OV	0.96	sc	48.21	OV	0.81	sc	40.94	OV	0.90	sc	45.10
	OF	0.76	sc	38.57	OF	1.00	sc	100.00	OF	0.74	sc	37.51	OF	1.00	sc	100.00
	OT	0.69	sc	34.73	OT	1.00	sc	100.00	OT	0.81	sc	40.69	OT	1.00	sc	100.00
6	OV	0.49	sc	24.42	OV	0.97	sc	48.72	OV	0.73	sc	36.65	OV	0.91	sc	45.53
	OF	0.56	sc	28.21	OF	0.94	sc	47.52	OF	0.59	sc	29.75	OF	1.00	sc	100.00
	OT	0.68	sc	33.90	OT	0.97	sc	48.43	OT	0.99	sc	49.51	OT	1.00	sc	100.00
7	OV	0.95	sc	47.55	OV	0.94	sc	47.24	OV	0.54	sc	27.13	OV	0.82	sc	41.29
	OF	0.20	sc	10.12	OF	0.88	sc	44.78	OF	0.75	sc	37.92	OF	1.00	sc	100.00
	OT	0.99	sc	49.72	OT	0.94	sc	46.95	OT	0.75	sc	37.64	OT	1.00	sc	100.00

**Table 3:** Average overlap per Test dataset.

Dataset nr.	OV			OF			OT			Avg. rank
	%	score	rank	%	score	rank	%	score	rank	
8	82.4	47.4	10.50	33.3	25.1	13.75	84.5	48.3	11.50	11.93
9	88.1	45.5	16.25	45.2	30.4	19.75	89.7	58.4	14.75	16.90
10	86.3	44.3	18.00	30.4	15.4	21.25	89.7	57.9	12.25	17.18
11	83.1	42.5	17.50	44.8	36.1	9.00	83.1	42.5	17.75	14.75
12	76.1	39.3	21.25	21.8	12.2	21.00	84.9	43.0	19.00	20.40
13	88.3	44.7	17.00	95.3	73.6	6.50	98.2	74.3	7.50	10.35
14	85.8	43.4	14.25	83.2	82.0	6.25	88.3	81.7	6.75	9.10
15	92.9	47.4	16.00	83.2	79.6	5.50	98.7	87.3	5.25	8.93
16	76.4	39.4	18.25	59.0	31.1	13.25	84.1	55.4	15.50	15.65
17	72.7	37.5	18.25	20.5	11.0	18.00	73.9	37.7	18.75	18.32
18	90.3	45.9	15.00	78.2	63.4	9.25	91.9	58.9	10.75	11.65
19	90.3	46.8	18.00	88.0	72.8	8.25	96.1	74.0	9.00	11.75
20	82.6	50.0	17.00	59.7	42.5	12.75	86.1	55.6	15.25	14.97
21	89.9	70.5	12.25	93.1	85.0	6.00	97.4	86.2	6.50	8.25
22	96.2	48.5	15.25	99.8	87.4	2.50	100.0	100.0	1.00	6.25
23	91.7	46.3	17.25	65.6	45.7	14.75	95.1	60.3	13.75	15.25
24	89.7	47.6	12.50	68.2	56.9	7.75	93.3	67.5	8.75	9.68
25	80.4	41.8	17.00	50.1	38.5	13.50	83.9	54.5	11.50	14.00
26	66.6	34.3	13.00	20.4	14.0	5.75	69.5	35.8	10.75	9.82
27	65.9	34.8	19.75	46.0	28.8	11.75	69.8	35.8	19.00	16.82
28	86.1	44.9	14.75	88.5	72.7	4.50	93.1	72.8	7.50	8.93
29	94.2	47.4	13.50	76.3	51.3	8.00	96.5	85.8	4.50	8.65
30	88.3	46.1	14.50	54.2	27.8	17.50	91.8	60.5	11.50	14.48
31	79.7	52.0	16.75	64.7	57.5	11.25	80.9	65.5	12.00	13.35
**Avg.**	**84.3**	**45.4**	**15.99**	**61.2**	**47.5**	**11.16**	**88.3**	**62.5**	**11.28**	**12.81**
**Std Dev**	**8.12**	**6.99**		**24.88**	**25.02**		**8.67**	**17.63**		

**Table 4:** Standard deviation and p-values for all parameters OV, OF, OT (Table 3).

	OV			OF			OT		
	average	std Dev	p_value	average	std Dev	p_value	average	std Dev	p_value
**%**	84.3333	8.1217	<.0001	61.2292	24.8783	0.0976	88.3542	8.6727	<.0001
**score**	45.3458	6.988	0.8106	47.5333	25.0234	0.6246	62.4875	17.6322	<.0001

**Table 5:** Average accuracy per Test dataset.

Dataset nr.	AI			Avg. rank
	mm	score	rank	
8	0.50	32.7	20.00	20.00
9	0.47	25.6	19.00	19.00
10	0.49	22.4	19.50	19.50
11	0.57	26.0	20.50	20.50
12	0.41	26.7	16.25	16.25
13	0.40	28.3	18.25	18.25
14	0.40	34.9	15.50	15.50
15	0.58	20.3	22.00	22.00
16	0.46	25.8	17.00	17.00
17	0.69	27.0	19.50	19.50
18	0.43	26.6	18.25	18.25
19	0.55	27.7	20.50	20.50
20	0.43	32.7	15.00	15.00
21	0.37	24.2	16.00	16.00
22	0.63	22.9	17.50	17.50
23	0.45	25.4	19.50	19.50
24	0.36	24.7	17.50	17.50
25	0.52	22.7	18.75	18.75
26	0.48	42.2	12.25	12.25
27	0.49	30.4	17.00	17.00
28	0.43	21.4	19.25	19.25
29	0.29	31.9	13.00	13.00
30	0.43	23.0	18.50	18.50
31	0.34	25.6	13.75	13.75
**Avg.**	**0.47**	**27.1**	**17.68**	**17.68**

**Table 6:** Summary for Test dataset.

Measure	% / mm			score			rank		
	min.	max.	avg.	min.	max.	avg.	min.	max.	avg.
OV	16.0%	100.0%	84.3%	8.7	100.0	45.4	1	24	15.99
OF	4.1%	100.0%	61.2%	2.1	100.0	47.5	1	24	11.16
OT	16.7%	100.0%	88.3%	9.1	100.0	62.5	1	24	11.28
AI	0.22 mm	0.98 mm	0.47 mm	13.5	55.8	27.1	5	24	17.68
**Total**							**1**	**24**	**15.24**

**Fig 10:**
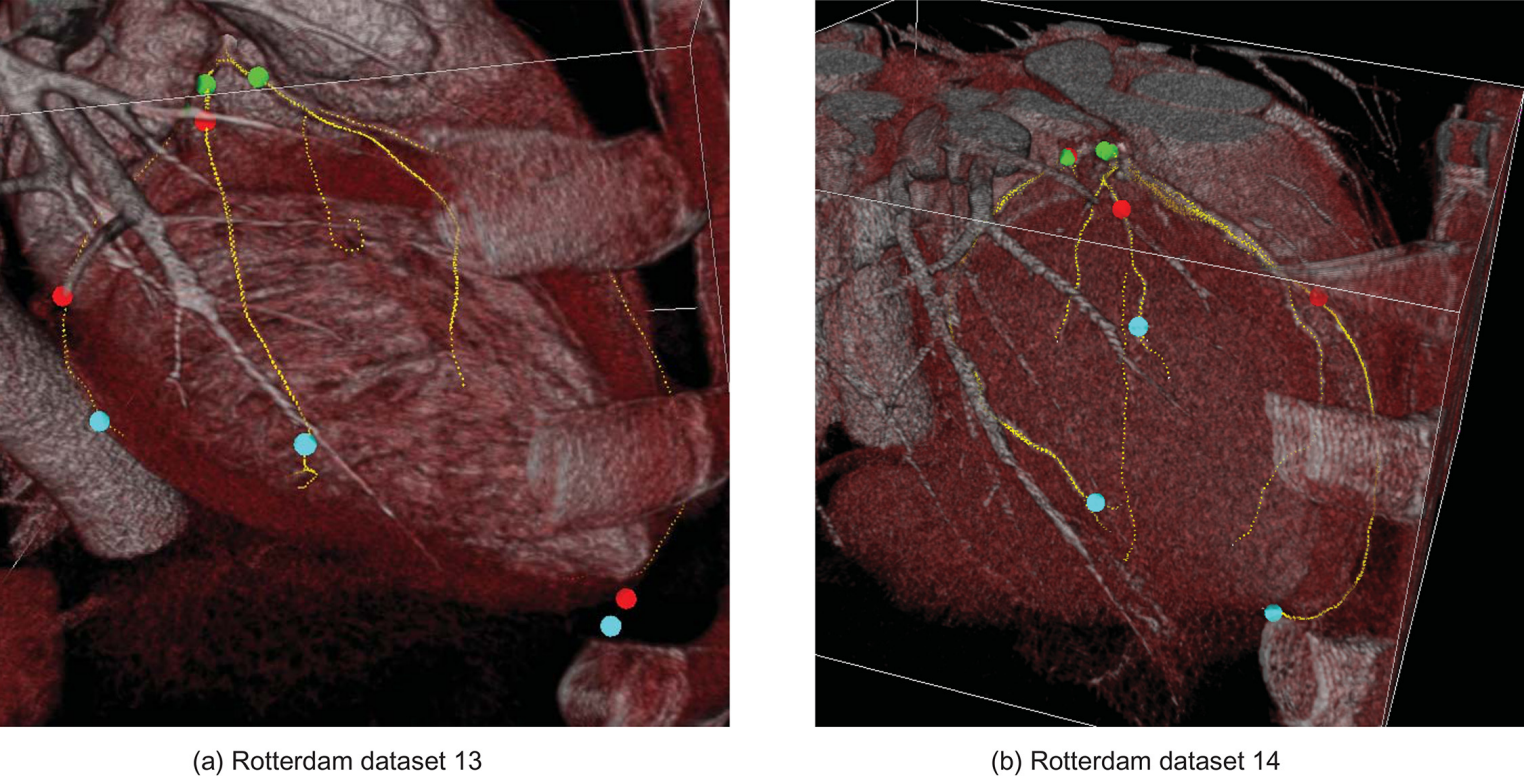
Examples of coronary artery structure found by the proposed method.

**Fig 11:**
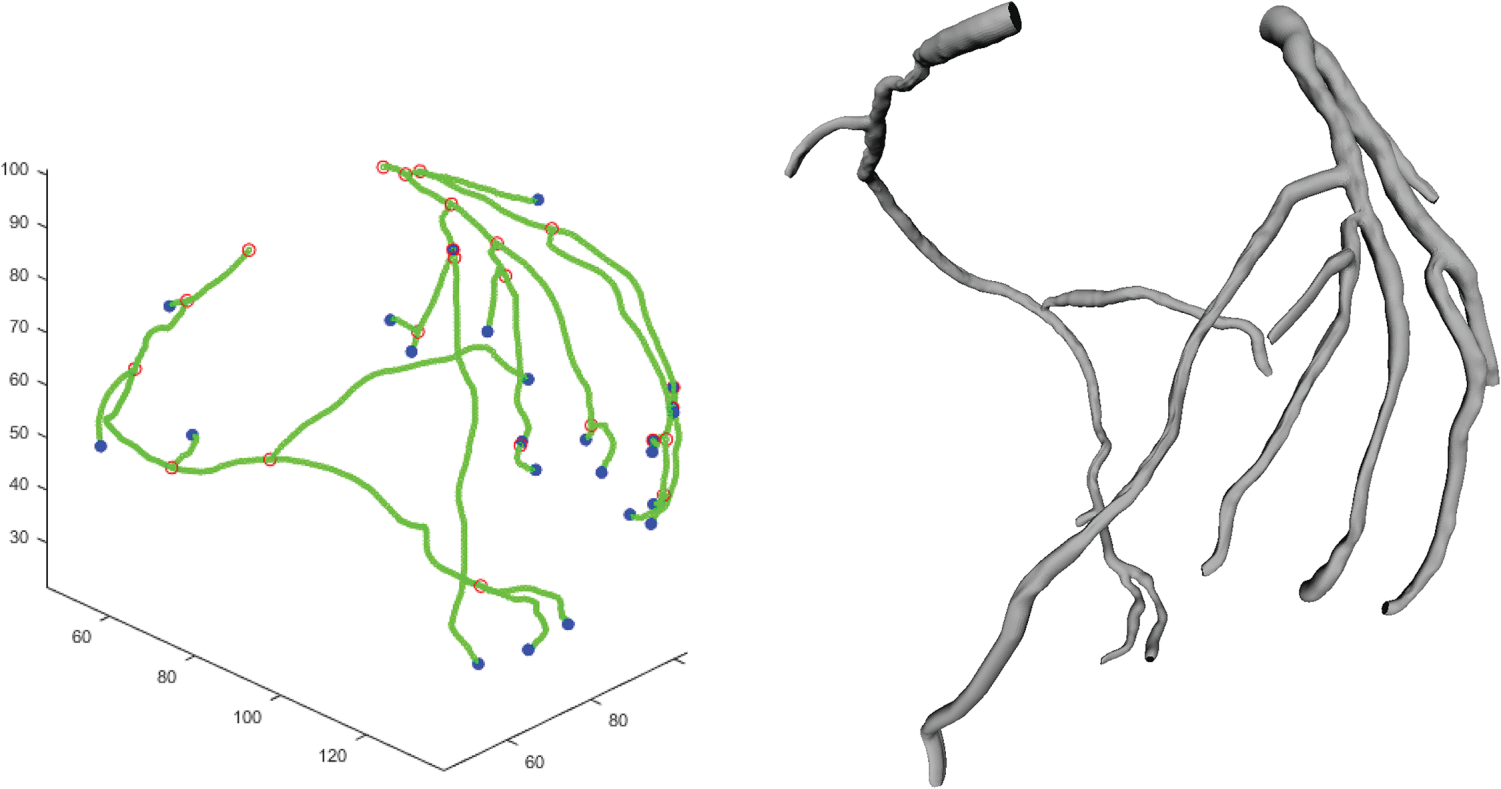
Example of centerline extracted (left) and coronary artery tree mesh reconstruction (right) found by the proposed method.

The running time is from 4 to 6 minute depending on complexity of each dataset. The proposed method finds the entire tree structure including small vessel branches as shown in Figs 10 and 11. In the first figure, the colored spheres represent reference points for the identification of selected vessels for the Rotterdam challenge. Fig 11 shows the reconstructed lumen boundary mesh which is to be used for CFFR (computed fractional flow reserve). For the generation of meshes, the lumen boundary surface is automatically optimized via surface constraint and image gradient as a post process.

In Tables 2–6, the scores and ranks for individual dataset are shown in detail. The rank is given from the score which is computed from non-linear increasing function of overlap ratio [26]. In Table 3, our Test dataset result is shown. In Table 4, it shows that the mean percentage values of OV and OT are significantly different (p < 0.0001).

For the comparison of challenge participating methods, the overlap scores of all the challenge participants (OV, OF, OT and average of them) are shown in Table 7. For the average overlap score, the median is 53.7 and ours is 51.8. The first and third quartiles are 47.1 and 63.2, respectively. Hence, interquartile range is 16.1. These statistics are obtained from the entire 24 challenge participants (methods) including us. However, three different competitions are mixed here: full automatic, given seed points and user-interactive. For fair comparison, we chose relevant 11 methods including us which are based on relatively similar condition (full automatic and one seed point at ostium and they are marked with a circle o at the last column of the table). Then the statistics becomes median 52.2 and the first and third quartiles are 43.7 and 55.8.

**Table 7:** Overlap Measure Scores for all the methods using Test dataset. Also accuracy distance measure is shown in **mm**. Last column is marked with a circle for the similar experiment to us.

participant ID	OV		OF		OT		aver. perc.	AI accu.	sel.
	%	score	%	score	%	score	%	mm	
MHT	98.5	84	83.1	72.8	98.7	84.5	93.4	0.23	
ShapeRegression	96.9	79.2	72.5	66.3	97.1	79.2	88.8	0.23	
ModelDrivenCenterline	93.5	53.4	76.5	54.9	95.6	70	88.5	0.2	o
VirtualContrast2b	96.7	73	74.5	63.3	96.9	74.7	89.4	0.27	
Tracer	95.1	71	63.5	52	95.5	70.2	84.7	0.26	
BayesianMaxPaths	97.5	81.6	78.8	70.8	97.7	81.5	91.3	0.29	
SupervisedExtraction	90.6	53.8	70.9	49	92.5	61.2	84.7	0.25	o
GFVCoronaryExtractor	93.7	55.9	74.2	52.9	95.9	68.5	87.9	0.3	o
DepthFirstModelFit	84.7	48.6	65.3	49.2	87	60.1	79.0	0.28	o
COR Analyzer	87.7	50.3	71.7	47.8	89.8	59.5	83.1	0.25	o
VesselTractography	96.4	64.3	69.9	51.6	97	70.3	87.8	0.36	
KnowledgeBasedMinPath	88.0	67.4	74.2	61.1	88.5	70	83.6	0.39	
GVFTube’n’Linkage	92.7	52.3	71.9	51.4	95.3	67	86.6	0.37	o
AutoCoronaryTree	84.7	46.5	59.5	36.1	86.2	50.3	76.8	0.34	o
CocomoBeach	78.8	42.5	64.4	40	81.2	46.9	74.8	0.29	o
TwoPointMinCost	91.9	64.5	56.4	45.6	92.5	64.5	80.3	0.46	
StatisticalTracking	81.5	45.5	59.1	46.7	84.6	59.5	75.1	0.51	o
VirtualContrast	75.6	39.2	56.1	34.5	78.7	45.6	70.1	0.39	o
AxialSymmetry	90.8	56.8	48.9	35.6	91.7	55.9	77.1	0.46	
TubSurfGradFlow	91.1	53.3	65.3	41.9	92.2	53.9	82.9	0.47	
ElasticModel	77	40.5	52.1	31.5	79	45.3	69.4	0.4	
3DInteractiveTrack	89.6	51.1	49.9	30.5	90.6	52.4	76.7	0.51	
CoronaryTreeMorphoRec	67.0	34.5	36.3	20.5	69.1	36.7	57.5	0.59	o
**Ours**	84.3	45.4	61.2	47.5	88.3	62.5	77.9	0.47	o
**Med. (all)**	**90.7**	**53.4**	**65.4**	**48.4**	**92.0**	**61.9**	**83.0**	**0.35**	
**Med. (sel.)**	**89.6**	**47.6**	**65.3**	**47.7**	**90.6**	**59.8**	**79.0**	**0.32**	

## Discussion

A new vessel tracking method based on stochastic geometric processes with active branch search is developed for 3-D coronary artery segmentation. The suggested method reconstructs the whole coronary artery tree structures with the estimation of the vessel lumen boundary which can immediately generate mesh of the coronary structure (Fig 11). The result of developed method is compared with the results of other methods as the centerline accuracy problem using Rotterdam coronary artery algorithm evaluation framework [26]. It shows high performance of OV 83.6%, OF 65.3% and OT 88.2% among sequential tracking methods which is not using other knowledge of vascular segmentation information. Our experiment result is comparable to most reported results in Rotterdam coronary artery algorithm evaluation framework [26]. In this Rotterdam coronary artery algorithm evaluation framework experiment results, a few show high percentages over 90% in OV measuring (also high scores). Such methods employ complicated hybrid methods using prior information like the left ventricle segmentation [30] (ModelDrivenCenterline in Table 7) or requires multiple processes [31] (GFVCoronaryExtractor). However, since our system is based on pure MAP (maximum a posteriori) estimation via vessel geometry model, we believe it is easy to adopting other techniques and can be combined for improvement. With regards to the accuracy distance measure number in Tables 5 and 7, our method shows rather large distances compared to other methods. We believe the reason is that our system concentrates on the reconstruction of the lumen wall instead of finding accurate centerlines and it does not optimize or adjust the centerlines after the first detection.

For the summary of newly developed active search scheme, it comes in three ways. The first one is for disconnected vessel (type 1) and the second is for disconnected branches (type 2). The third one is using branch occurrence model (type 3). For the disconnected vessel the search is straightforward and just tries to find the vessel segment over the gap. Then it connects them. And for the missing branches, both high probabilistic region investigation (type 2) and the branch occurrence model (Poisson) are employed (type 3).

Such active search method plays an important role as follows. Especially when there is a local discontinuity in vessel image, newly suggested active search method overcomes difficulties due to the nature of sequential vessel tracking techniques. In CCTA images, vessel sometimes can be seen to be disconnected due to plaque or noise in the image. In such cases, the active search method will overcome the seemingly disconnected gap. In addition, such discontinuity frequently occurs near branch points. When there are bifurcations, there can be blood turbulence at the bifurcation area and plaque can be easily built-up [21]. Such plaque can makes vessel branch seem to be disconnected. This is why the type 2 active search is employed. This is an improvement to conventional branch detection and vessel finding algorithm since it provides an efficient local search scheme. Benefiting from the new active search scheme, the proposed method has high capability of finding seemingly disconnected vessel and branches.

For the termination of vessel tracking, a newly devised practical planar vesselness measure is used. It is computationally efficient and comparable to popular vesselness measure [28] as shown in Fig 8. More recently, a new deep neural network learning based vessel classifier is devised and tested. It is based on a convolutional neural network (CNN) and a preliminary test shows a high classification rate in distinguishing vessels from non-vessel substances [32].

As for statistical branch occurrence location model, it is used only for the first branch of the left main artery for now but the complete statistical branch location model for the entire coronary artery system is under study. More analysis for the active search is required. After the entire tree structure is found, the lumen mesh is optimized via surface constraint and gradient information and this mesh is to be used for other tasks (see Fig 11). Also, automatic aorta and ostium segmentation is under development now and the system will be full automatic without requiring starting points.
